# *Akkermansia muciniphila* Alleviates *Porphyromonas gingivalis-*induced Periodontal Disease by Enhancing Bacterial Clearance

**DOI:** 10.1007/s12602-025-10541-2

**Published:** 2025-04-29

**Authors:** Qin Hu, Wai Keung Leung, Aneesha Acharya, Xuan Li, George Pelekos

**Affiliations:** 1 Faculty of Dentistry, The University of Hong Kong, 34 Hospital Road, Sai Ying Pun, Hong Kong SAR, China; 2 Dr D Y Patil Dental College and Hospital, Pune, India

**Keywords:** *Akkermansia muciniphila*, *Porphyromonas gingivalis*, Periodontal disease, Host-microbe interaction, TLR-MyD88

## Abstract

This study is to investigate the role of *Akkermansia muciniphila* (*Am*) in enhancing immune defense against *Porphyromonas gingivalis* (*Pg*)-induced periodontal disease. Twenty C57BL/6 J mice received 50 µL of *Pg* suspension (1.5 × 10^9^ CFU/mL) with or without 50 µL of *Am* suspension (1.5 × 10^9^ CFU/mL) orally every 2 days for a total of 18 administrations to assess bone resorption and inflammation. Gingival cervical fluid and periodontal plaques were collected for microbiota analysis using 16S sequencing. THP-1 (a human leukemia monocytic cell line) differentiated macrophages were used to explore the underlying beneficial mechanisms of *Am* by evaluating gene expression, cytokine production, and phagocytosis activity. *Am* administration attenuated alveolar bone loss and reduced inflammation in *Pg*-induced periodontitis in mice. Microbiota analysis revealed that *Am* reduced bacterial load and modified the composition of periodontal microbiota. In THP-1 macrophages, *Am* enhanced the phagocytosis of *Pg* by restoring MyD88 protein levels. RNA sequencing and western blotting results showed that *Am* upregulated TLR2 and MyD88 expression while downregulating C5aR, indicating interference with the TLR2-C5aR-MyD88 interplay. *Am* enhances immune defense against *Pg*-induced periodontal disease by modulating the TLR2-C5aR-MyD88 signaling pathway. These findings suggest that *Am* could be a promising therapeutic option for managing periodontal disease.

**Supplementary Information:**

The online version contains supplementary material available at 10.1007/s12602-025-10541-2.

## Introduction

Periodontitis is a chronic inflammatory condition that arises from microbial dysbiosis and progresses through the destruction of the supporting structures around teeth, ultimately leading to tooth loss. It affects 45–50% of the global population and is associated with systemic conditions like cardiovascular diseases, diabetes, and Alzheimer’s disease [1–3]. Restoring the periodontal microbiota from dysbiosis to symbiosis is crucial for preventing or treating periodontal diseases and related health issues.

*Porphyromonas gingivalis* (*Pg*), a Gram-negative anaerobic bacterium, is a keystone pathogen of periodontitis [4, 5]. For instance, *Pg* produces gingipains, which can degrade the cytokines/chemokines to paralyze the immune system, digest tight junction proteins of the epithelium to damage barrier function, and cleave macrophage receptors to evade phagocytosis [6, 7]. Additionally, *Pg* can exploit the Toll-like receptors (TLR) and complement receptors to degrade MyD88, a protective mechanism of the host, and disrupt the host’s immune response [8]. These virulence factors allow *Pg* to evade immune surveillance, induce microbial dysbiosis in the periodontal environment, leading to disease progression. Current management approaches, such as scaling, root planing, antibiotics, and antimicrobial mouthwash, have drawbacks like tooth sensitivity, introducing bacteria into the bloodstream, and drug resistance [9]. In light of these challenges, leveraging the immune system and restoring its protective actions subverted by *Pg*, is a promising alternative to tackle *Pg* and treat *Pg*-induced periodontal disease.

Probiotics have shown immunomodulatory characteristics, making them attractive therapeutic options for various health conditions [10, 11]. *Akkermansia muciniphila* (*Am*), a Gram-negative anaerobic commensal colonizer in the human gut, has emerged as a next-generation probiotic. The reduction or absence of *Am* is tightly linked to various diseases, including cancers, metabolic disorders, and inflammatory diseases [12–14]. Several studies also reveal that the administration of live or pasteurized *Am* can alleviate periodontitis while the underlying mechanisms remain to be clarified [15–17].

We hypothesized that *Am* benefits periodontal health through enhancing host defensive immunity and modulating periodontal microbiota. To explore this, we examined the role of *Am* in *Pg*-induced periodontitis in mice. Next, the underlying mechanisms were determined in THP- 1 (a human leukemia monocytic cell line) differentiated macrophages.

## Methodology

### Bacterial Strains and Culture Conditions

*A. muciniphila* (*Am*, BAA- 835) and *P. gingivalis* (*Pg*, W83) were obtained from ATCC (USA). *Am* was cultured on brain infusion agar plates, while *Pg* was grown anaerobically at 37 °C on blood agar plates. *Pg* was also cultured in tryptic soy broth (TSB) and *Am* in brain heart infusion (BHI) broth with 0.3% mucin. Pasteurized *Am* (*pAm*) was prepared by suspending *Am* in phosphate-buffered saline (PBS), pasteurizing at 70 °C for 30 min, and storing at − 80 °C. Log-phase bacterial cells were collected by centrifugation, resuspended in media or PBS to an OD of 0.1 (1.5 × 10^7^ CFU/mL), and used as needed. Bacterial supernatants were filtered and stored at − 80 °C.

### Animals

A total of 20 C57BL/6 J mice were primed with prophylactic antibiotic treatment using sulfamethoxazole (700 µg/mL) and trimethoprim (400 µg/mL) in water bottles for 7 days, followed by a 3-day rest [18]. The mice were then divided into four groups: (1) the control group received 50 µL of 1% carboxymethyl cellulose (CMC) solution; (2) the *Pg* group received 50 µL of *Pg* suspension (CMC solvent) at a concentration of 1.5 × 10^9^ CFU/mL; (3) the *Am* group received 50 µL of *Am* suspension (CMC solvent) at a concentration of 1.5 × 10^9^ CFU/mL; (4) the mixture group received 50 µL of a combined *Am* and *Pg* suspension (CMC solvent), each at 1.5 × 10^9^ CFU/mL. Solutions were administered to the mice orally using a flat-tipped syringe every 2 days, totaling 18 administrations. After the bacterial challenge, the mice underwent a 5-day washout period.

To explore the impact of *Am*, gingival cervical fluid (GCF) was collected from the first right upper molar at days 15, 30, and 45 to assess the cultivable bacterial loads. On the final day of the study, GCF was collected again to analyze the microbial community structure and composition. The process was conducted in a clean cabinet using sterile paper points (size #15). The paper points were gently placed in both the buccal and palatal crevices of each first molar for 10–15 s. The paper tips with GCF were then dissected using sterile scissors. For bacterial culture, GCF-loaded the paper tips were suspended in PBS solution.

For 16S sequencing, the collected paper tips were immediately frozen in liquid nitrogen. The sequencing data were processed and analyzed under the R environment. The clean 250bps paired-end reads were trimmed, filtered, and merged into amplicon sequence variants (ASVs) using the DADA2 pipeline [19]. The taxonomy of ASVs was assigned by referring to the NCBI 16S RefSeq database. Beta diversity was assessed by Bray–Curtis dissimilarity using phyloseq. For functional analysis, ASVs were assigned to KEGG terms by the Picrust2 pipeline [20]. Differential species and KEGG terms were determined by using limma-voom pipeline [21]. The resulting differential species were summarized in the Venn diagrams using ggvenn. The dot plot of differential KEGG terms was plotted using ggplot2. All *p*-values of multiple tests were adjusted by the Benjamini–Hochberg method. The alpha of the statistical test was set at 0.05.

All mice were sacrificed on the last day. The upper right jaws were carefully dissected for micro-CT analysis. Based on the sample characteristics, the scanning parameters were set to a voltage of 50 kV, a current of 500 µA, and a resolution of 8.7 µm. All projection images obtained from the scan were reconstructed into tomographic images using NRecon software (v.1.7.1.0). Using DataViewer software (v.1.5.6.2), the coronal, sagittal, and transverse planes of the sample’s tomographic images were aligned. The distance from the alveolar bone crest between the second and third molars to the cementoenamel junction of the first molar was measured. Centered on the cross-section of the molar, a square area of 1.2 × 1.2 mm was selected, excluding the dental tissue. The height ranged from the root bifurcation to the apex, defining the region of interest (ROI). The selected ROI of the alveolar bone was analyzed using CTAn software (v.1.20) to calculate parameters such as bone volume fraction (BV/TV). Finally, the three-dimensional structure of the entire jawbone was visualized using CTVox software (v.3.3.0).

The upper left jaws were fixed, decalcified, embedded in paraffin, and sectioned for immunohistochemical staining. Immunohistochemical staining was performed using the following primary and secondary antibodies: IL- 6 (Catalog No: ab290735, Abcam, UK) at a dilution of 1:200, IL- 1β (Catalog No: ab283818, Abcam, UK) at a dilution of 1:200, MCP- 1 (Catalog No: ab25124, Abcam, UK) at a dilution of 1:200, MPO (Catalog No: 22225–1-AP, Proteintech, USA) at a dilution of 1:200, and goat anti-rabbit IgG H&L (HRP) (Catalog No: ab6721, Abcam, UK) at a dilution of 1:1000. For tartrate-resistant acid phosphatase (TRAP) staining, slides were incubated with TRAP staining solution mix (Servicebio, CN) according to the manufacturer’s instruction.

### Cell Culture

THP- 1 cells (ATCC, USA) were maintained in RPMI- 1640 medium (ATCC, USA) containing 10% fetal bovine serum, 100 µg/mL normocin (InvivoGen, USA) and 50 µM β-mercaptoethanol, and the cells were differentiated into macrophages with phorbol- 12-myristate- 13-acetate (PMA, Sigma-Aldrich, USA) at 100 ng/mL for 24 h followed by at least a 24-h rest in PMA-free medium prior to assays. Cells were cultured at 37 °C in a 5% CO_2_ atmosphere.

### Cytocompatibility and Bacterial Administration Protocol for *In Vitro* Assays

THP- 1 differentiated macrophages were seeded in 6-well plates and cultured overnight. The cells were then exposed to *Am* at a varying multiplicity of infection (MOI). After the bacterial infection, the metabolic activity of the cells was evaluated using Cell Counting Kit- 8 (CCK- 8, Dojindo Laboratories, Japan), while plasma membrane integrity was assessed using CyQUANT LDH cytotoxicity assay kit (Thermo Fisher Scientific, USA). Meanwhile, cell viability was determined using the LIVE/DEAD viability/cytotoxicity kit (Invitrogen, USA).

Referring to the following assays, the treatment groups were established as follows: Ctrl (medium alone), *Am* (MOI: 100), *Pg* (MOI: 10), mixture (mixture of *Am* at MOI of 100 and *Pg* at MOI of 10), *pAm* (MOI: 100), and *pAm* + *Pg* (mixture of *pAm* at MOI of 100 and *Pg* at MOI of 10).

### Effect of Am on the Adhesion and Cellular Internalization of Pg

THP- 1 was differentiated by PMA in 6-well plates at a density of 1 × 10^6^ cells/well and pre-treated with/without *Am* or *pAm* for 3 h at an MOI of 100. Gentamicin (GTM) and ampicillin (AMP) were used to eliminate *Am* interference in CFU counting. Cells were infected with *Pg* for 1 h, washed with PBS, and treated with GTM and metronidazole (MTZ) to assess adherent and internalized *Pg*. Cells were lysed, serially diluted, and plated for CFU counting. For MyD88-associated phagocytosis, THP- 1 macrophages were infected with *Pg*, *Pg* with *Am*, or *Pg* with *Am* and MyD88 inhibitor (TJ-M2010 - 5) for 3 h. Cells were collected, stained with mouse anti-*Pg* primary antibody followed by Alexa Fluor 488-conjugated anti-mouse IgG secondary antibody, and analyzed for intracellular *Pg* using a BD LSRFortessa flow cytometer and FlowJo software.

### Enzyme-linked Immunosorbent Assay (ELISA) and Cytokine Array Assay

Supernatants of cells challenged by different bacteria were collected and debris removed by centrifugation. Cytokine or chemokine levels were quantified using ELISA kits (R&D Systems, USA). Culture media from bacteria-infected THP- 1 cells were tested using the Proteome Profiler Human XL cytokine array kit ARY022B (R&D Systems, USA). Array panels were visualized using the iBright 1500 (Invitrogen, USA) and analyzed with HLImage +  + (Western Vision Software, USA).

### Real-Time Quantitative Transcription PCR (RT-qPCR), and RNA Sequencing

Total RNA was extracted with RNAfast200 kit (Fastagen, China). RNA was reverse transcribed into cDNA with the TB Green® Premix DimerEraser™ Kit (Takara, Japan). Quantitative PCR (qPCR) was performed on the ABI Prism 7700 (Applied Biosystems, USA). The primer sequences used were listed in Table S1. Relative gene expression levels were normalized to endogenous reference genes *ACTB* with the comparative threshold cycle (2^−△△ct^) method. RNA sequencing was conducted on RNA from THP- 1 cells treated with *Am* and/or *Pg*. Samples were tested using the DNBSEQ platform, and differential expression was calculated using DESeq2.

### Western Blotting

Cells were lysed using M-PER reagent with Halt protease and phosphatase inhibitors. Lysates were centrifuged, and protein concentration was measured using the Pierce BCA protein assay kit. Proteins were denatured, separated on 10% SDS polyacrylamide gels, and transferred to Amersham Hybond P membranes. Membranes were blocked with 5% BSA for 1 h at room temperature. They were then incubated overnight at 4 °C with rabbit monoclonal primary antibodies: anti-MyD88 antibody from Cell Signaling Technology and anti-C5aR antibody from Proteintech, both at a dilution of 1:1000. After washing, membranes were incubated with HRP-conjugated secondary antibodies (abcam). Blots were detected using the WesternBright Sirius kit and imaged with the ChemiDoc XRS + System. Images were processed using ImageJ. The protein bands were selected using the “rectangular” tool, ensuring the same frame size across all lanes. The density of the blots was measured and recorded using ImageJ. Before ANOVA analysis, the results for the target protein were then normalized to each corresponding Actin/GAPDH value.

### Statistical Analysis

All experiments were repeated at least three times independently. The one-way analysis of variance (ANOVA) with Tukey’s multiple comparisons test was used for statistical evaluation of all results. A *p*-value < 0.05 was considered statistically significant.

## Results

### Am Attenuated the Bone Resorption and Inflammation in Pg-Induced Periodontitis

**Fig. 1:**
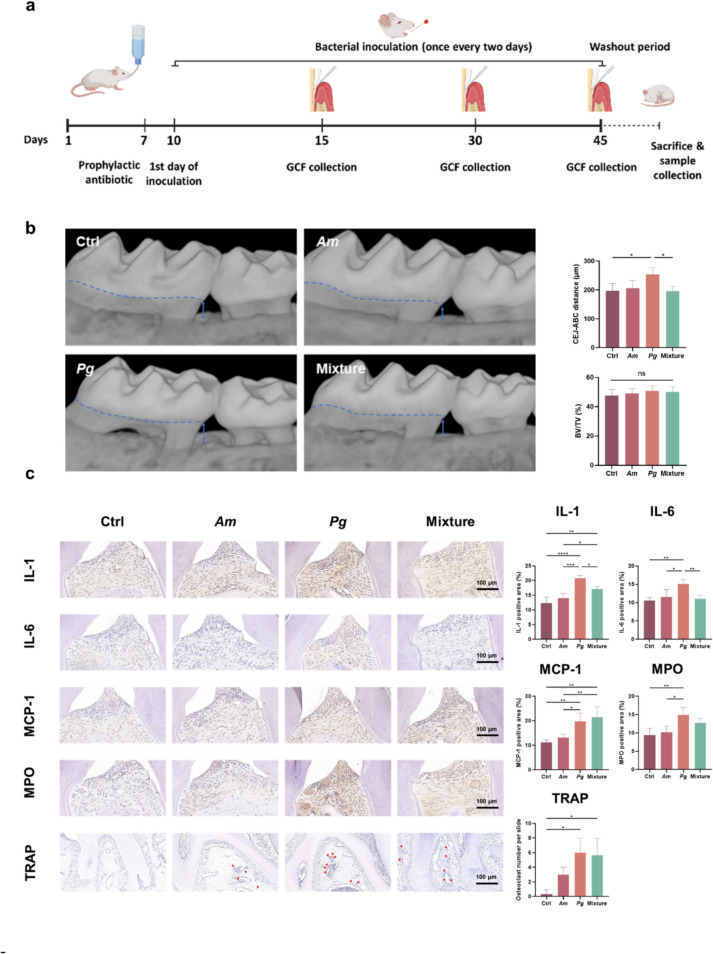
*Am* mitigated *Pg*-initiated periodontitis. **a** Experimental design: specific-pathogen-free mice received prophylactic antibiotics for 7 days with a 3-day rest. Starting from day 10, mice received different treatments (Ctrl, *n* = 5, receiving CMC; *Am*, *n* = 5, receiving *Am*; *Pg*, *n* = 5, receiving *Pg*; Mixture, *n* = 5, receiving a mixture solution of *Am* and *Pg*), repeated every 2 days until day 45. Gingival crevicular fluid (GCF) was collected on days 15, 30, and 45. After a 5-day washout period, plaques surrounding the periodontal area were collected for 16S sequencing, and mice were sacrificed for sample collections. **b** Micro-CT images of the right upper molar periodontal sections. The cementoenamel junction-alveolar bone crest (CEJ-ABC) distance and bone volume over total volume (BV/TV) fraction were analysed. **c** Histological examination of the left upper molar periodontal sections, with immunohistochemical staining for IL- 1, IL- 6, MCP- 1, MPO, and TRAP. Data are presented as mean ± SD. *p < 0.05, **p < 0.01, ***p < 0.001, and ****p < 0.0001 versus the control and matched groups

Figure 1a illustrates the scheme of animal study. Micro-CT results showed that mice treated with *Pg* exhibited a significant increase in the cementoenamel junction-alveolar bone crest (CEJ-ABC) distance compared with the control group (*p* = 0.0372), while the addition of *Am* attenuated the resorption (Fig. 1b). However, the BV/TV fraction (bone volume relative to total tissue volume) showed no statistical difference across the four groups. Immunohistochemistry (IHC) staining showed inflammatory changes in the gingival tissues after 45 days of treatment (Fig. 1c). Compared with the control group, administration of *Pg* stimulated remarkable IL- 1 (*p* < 0.0001), IL- 6 (*p* = 0.0019), MCP- 1 (*p* = 0.006), and myeloperoxidase (MPO, *p* = 0.0034) expression. However, *Am* did not increase their expression. The mixture group showed a less potent inflammatory response compared to *Pg* group with significantly reduced expression in IL- 1 (*p* = 0.0192) and IL- 6 (*p* = 0.0044). An increase in TRAP-positive cells was observed in both the *Pg* (*p* = 0.012) and mixture (*p* = 0.0167) groups, indicating elevated osteoclast activity.

### Am Reduced Bacterial Load and Modified the Microbiota Composition in Periodontitis

**Fig. 2:**
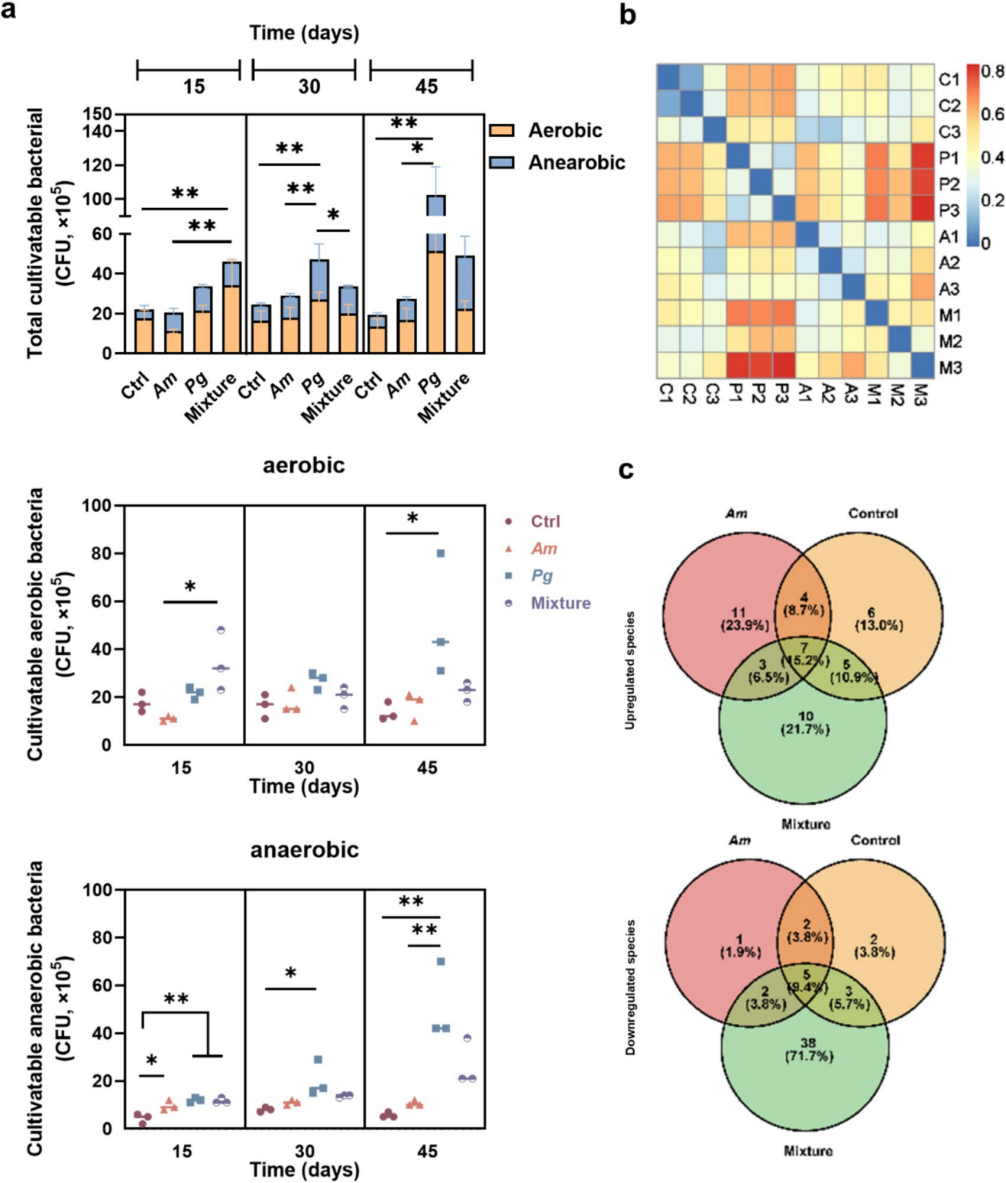
*Am* modified periodontal microbiota during periodontitis. **a** Bacterial load (total, aerobic, and anaerobic culturable bacteria) in the GCF from different groups at three-time points. **b** Beta-diversity heatmap representing the Bray–Curtis dissimilarity in bacterial community structure among different treatment groups. Each cell in the heatmap corresponds to the dissimilarity value between the two samples. **c** Venn diagrams showing the number of upregulated (up) and downregulated (down) species observed in different groups compared to the *Pg* group. Data are presented as mean ± SD. *p < 0.05 and **p < 0.01 versus the control and matched groups

An overall increasing trend was observed for both aerobic and anaerobic bacteria load in the GCF from the group inoculated with *Pg* (Fig. 2a), while the control group showed a steady bacterial count over time. After 15 days, the group treated with *Am* displayed a rise in the count of anaerobic bacteria. *Pg* induced notable increases in both aerobic and anaerobic bacterial load over time. The introduction of the bacterial mixture only resulted in an elevation of the bacterial load on day 15. As the treatment time extended, the bacterial load in the mixture group showed no statistically significant difference compared to the control group.

**Fig. 3:**
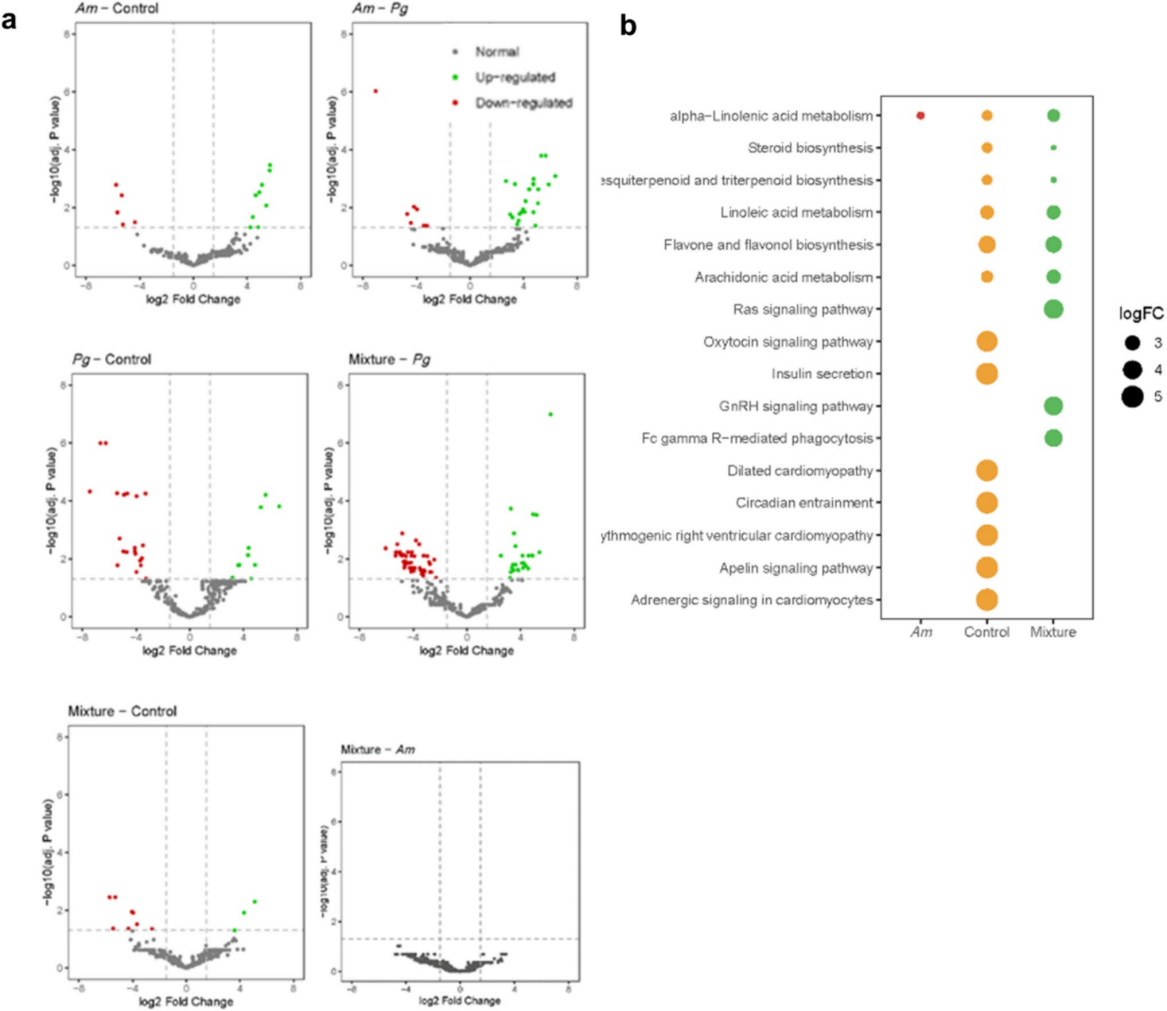
The impact of *Am* on the microbial composition and function. **a** Volcano plots showing the differential abundance of ASVs due to the treatment effect. Each point corresponds to an individual ASV. The *x*-axis position represents the abundance fold change. The dashed line indicates the threshold for significant differential ASVs (|log2(FC)|> 1.5). Green dots, red dots, and grey dots represented significantly enriched ASVs (upregulated), significantly depleted ASVs (downdownregulated), and no difference ASVs, respectively. **b** Bubble plot representing the differences in the abundance (%) of potential functions (KEGG) compared with the *Pg* group

Regarding the 16S sequencing result, the heat map in Fig. 2b showed the similarities among different samples, where the microbiome composition from the *Pg* group significantly deviated from that of the control and mixture groups. When compared to the *Pg* group, seven species were upregulated (*Prevotellamassilia timonensis*, *Muribaclum intestinale*, *Akkermansia muciniphila*, *Faeclicatena orotica*, *Duncaniella freteri*, *Acetatifactor muris*, *Faecalicatena fissicatena*) and five were downregulated ([*Eubacteriuim*] *sulci*, *Succiniclasticum ruminis*, *Slackia heliotrinireducens*, *Pyramidobacter piscolens*, *Mailhella massiliensis*) across the remaining three groups (Fig. 2c). Among them, those upregulated species are common commensals in the host microbiome, while the downregulated species are all anaerobic bacteria, most of which are associated with various disease conditions [22, 23]. The microbiomes of the *Am* and mixture groups exhibited no difference in the amplicon sequence variants (ASVs) in the differential abundance analysis (Fig. 3a). The bubble plot indicated that the microbial function of all other three groups varied from that of the *Pg* group in the alpha-linolenic acid metabolism function, which is significantly related to the MyD88/NF-κB pathway (Fig. 3b) [24].

### The Impact of Am on the Immune Response of THP- 1 Differentiated Macrophages

Macrophages play a crucial role in bacterial clearance during infections. In this study, we evaluated the cytocompatibility of *Am* with THP- 1-derived macrophages at various infection ratios (Fig. S1a). A decrease in viability was observed in THP- 1-derived macrophages from the infection ratio of 500:1. Consequently, for subsequent in vitro experiments, the MOI for *Am* on THP- 1 cells was standardized to 100:1.

**Fig. 4:**
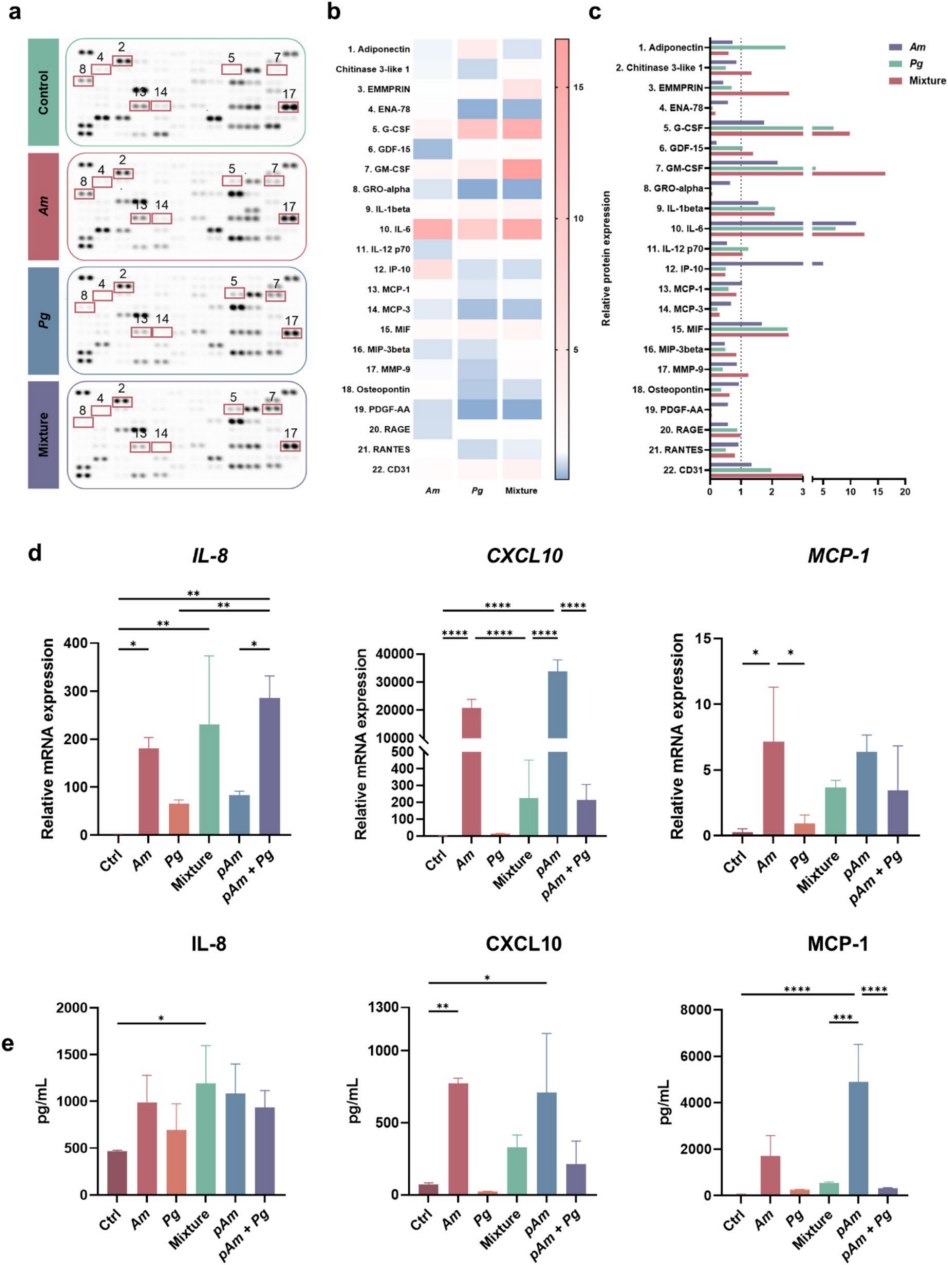
*Am* facilitated the immune response of THP- 1. **a**, **b**, **c** THP- 1-differentiated macrophages were treated with *Am*, *Pg*, or their combination for 24 h, respectively. The supernatants were analyzed using the Proteome Profiler Human XL Cytokine Array Kit. Twenty-two out of 105 proteins showing the greatest differences were highlighted in the membranes, analyzed, and presented in the heatmap and bar chart. **d** THP- 1-differentiated macrophages were treated with live or pasteurized *Am* with or without *Pg* for 6 h to evaluate the transcription levels of IL- 8, CXCL10, and MCP- 1. **e** Supernatants were collected from THP- 1 differentiated macrophages at 24 h for the ELISA assays to evaluate the protein levels of IL- 8, CXCL10, and MCP- 1. Data are presented as mean ± SD. *p < 0.05, **p < 0.01, ***p < 0.001, and ****p < 0.0001 versus the control and matched groups

The cytokine array results indicated that 22 out of the 105 analyzed cytokines exhibited notable intensity changes across different groups, as highlighted on the array membranes (Fig. 4a). The expression differences of these proteins were further presented in both a heat map and a bar chart (Fig. 4b, c). *Am* stimulated the expression of cell recruitment and accumulation-related cytokines, including ENA- 78, GRO-alpha, MCP- 1, and MCP- 3, while *Pg* had the opposite effect. Moreover, MMP- 9, which is associated with cell invasion and migration and plays a crucial role in host immune function, was decreased by *Pg* compared with the control group [25, 26]. G-CSF, as a key regulator of neutrophils, was enhanced by the stimulation of *Am*, *Pg*, and their combination. A similar expression pattern was also observed in GM-CSF, which is essential in the anti-infection process. Furthermore, chitinase- 3-like protein 1, which is induced during infection and promotes bacterial clearance, was suppressed in the *Pg* group [27, 28]. These results suggest a possible mechanism for how *Pg* evades the host immune response and reveal the potential of *Am* to restore immune function. In addition, the expressions of MCP- 1, IL- 8, and CXCL10 were quantitively evaluated at gene and protein levels in the cells after different bacteria stimulation (Fig. 4d, e). *Am* or *pAm* could increase the expression of MCP- 1, IL- 8, and CXCL10. Although *Pg* alone showed subtle changes to THP- 1, the tested chemokine levels could be upregulated to some extent by adding *Am* or *pAm*. These results demonstrated that *Am* or *pAm* could stimulate acute inflammation perturbed by *Pg* and enhance the function of immune cells.

### Am Enhanced the Phagocytosis of THP- 1 Derived Macrophages

**Fig. 5:**
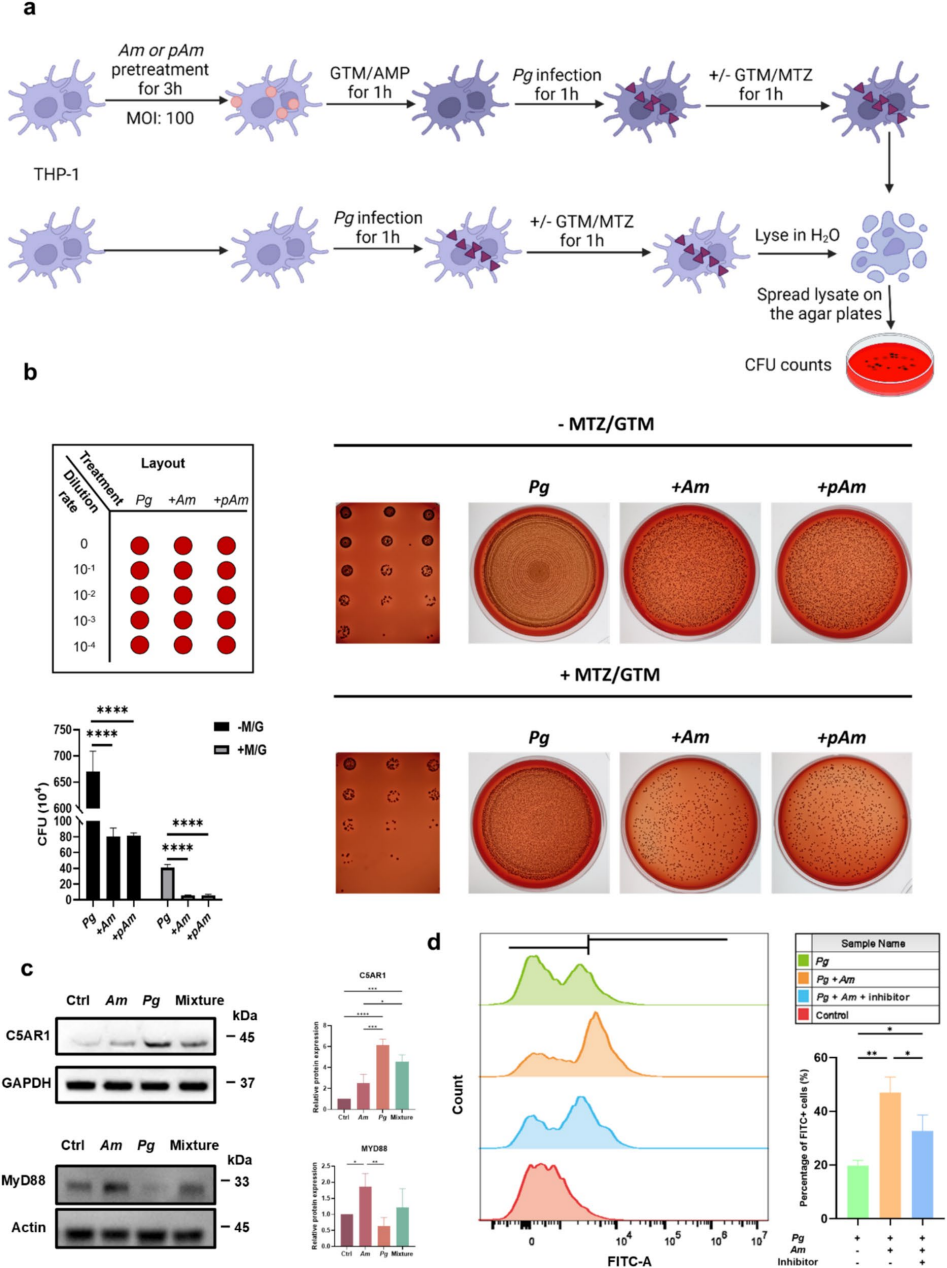
*Am* enhanced the phagocytosis of THP- 1 via MyD88. **a** Infection protocol. PMA-differentiated THP- 1 cells were assigned to two groups. One group was pretreated with *Am*/*pAm* at the MOI of 100:1. Cells then were treated with GTM (0.5 mg mL^−1^)/AMP (0.2 mg mL^−1^) solutions to remove *Am* followed by *Pg* infection at the MOI of 10:1 for 1 h. Cells were treated with/without GTM/AMP to differentiate the adherent *Pg* and intracellular *Pg*. The other group was infected with *Pg* followed by the same procedures. Cells were lysed in water and lysate was plated on blood agar plates for CFU counting. **b** Live or pasteurized *Am* pretreated THP- 1 macrophages were infected with *Pg*. Cells were rinsed with or without MTZ and GTM to eliminate the adherent *Pg* followed by lysis in H_2_O. Lysates were plated on blood agar plates and incubated in anaerobic conditions for 7 days to count the colony-forming units of *Pg*. **c** Western blot analysis of C5 AR1 and MyD88 protein level in THP- 1 cells treated with different bacteria (*Am* at the MOI of 100, *Pg* at the MOI of 10) or their combination for 3 h. **d** Flow cytometry analysis of the intracellular *Pg*. THP- 1 cells were treated with *Pg*, *Pg* + *Am*, or *Pg* + *Am* + MyD88 inhibitor (10 µM) for 3 h and then fixed. *Pg* was detected with a specific anti-*Pg* antibody in the flow cytometry assay. Data are presented as mean ± SD. *p < 0.05, **p < 0.01, ***p < 0.001, and ****p < 0.0001 versus the control and matched groups

A schematic illustration of the treatment procedure is shown in Fig. 5a. The results showed that the amount of adhesive and invasive *Pg* decreased in the *Am* or *pAm* pre-treated group (*p* < 0.0001, Fig. 5b). Additionally, even after a 1-h disinfection by MTZ/GTM to eliminate the attached *Pg* on the cell surface, the pre-treatment of *Am* or *pAm* could still significantly reduce the amount of intracellular *Pg* (*p* < 0.0001). A previous study revealed that *Pg* induced MyD88 degradation through the interplay between TLR2 and C5aR which in turn caused the disengagement of *Pg* from bacterial clearance [8]. The protein levels of C5 AR1 and MyD88 in different groups were further determined (Fig. 5c). *Pg* elevated the expression of C5 AR1 compared to control group (*p* < 0.0001) while the addition of *Am* relatively diminished *Pg*’s effect. One the other hand, *Pg* tended to degrade the MyD88 protein whereas *Am* was capable of increasing and restoring the protein levels. The flow cytometry result in Fig. 5d showed that *Am* could significantly enhance the internalization of *Pg* by the THP- 1 cells (*p* = 0.0013). Yet, the administration of MyD88 inhibitor could reduce the intracellular *Pg* (*p* = 0.0285), suggesting that *Am* mediated the phagocytosis of *Pg* by THP- 1 via the TLR-C5aR-MyD88 pathway.

### Am Interfered the TLR-C5aR-MyD88 Interplay

**Fig. 6:**
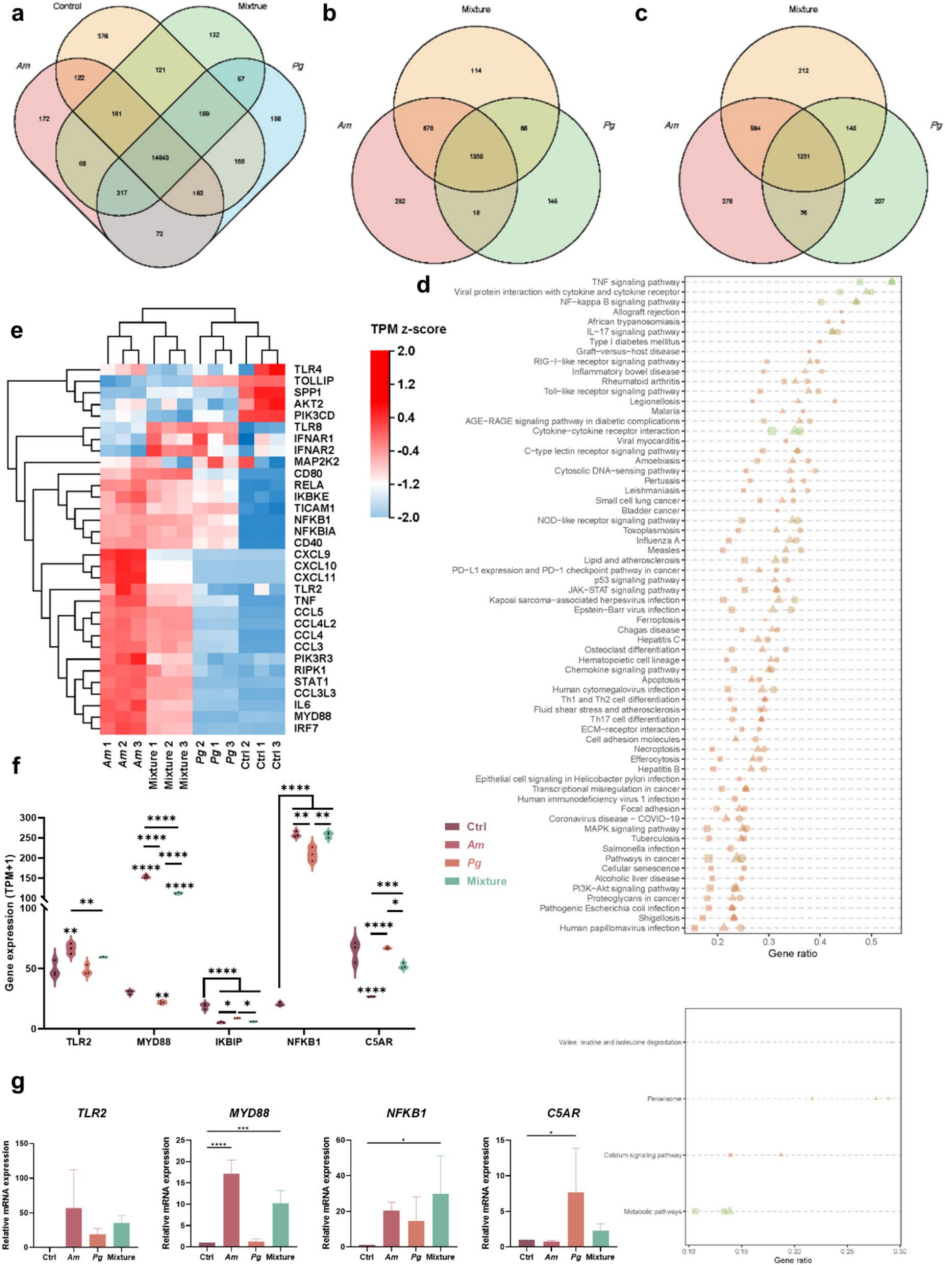
*Am* interefered the TLR-C5aR-MyD88 interplay. **a**, **b**, **c** Venn diagram showing the distribution of genes identified. Upregulated (left) and downregulated (right) genes were shown in the Venn diagrams. **d** The up- and downregulated genes were called to KEGG term separately and plotted. **e** Cluster analysis of the gene expression in the KEGG TLR signal pathway. RNA was extracted from THP- 1 cells treated by bacteria for 6 h. Different colors indicate the expression intensity: red represents higher expression, and blue represents lower expression. **f** Selected differentially expressed genes. **g** Relative related gene expression validated by RT-PCR. Data are presented as mean ± SD. *p < 0.05, **p < 0.01, ***p < 0.001, and ****p < 0.0001 versus the control and matched groups

For RNA sequencing, a total of 16,843 genes were identified. The Venn diagram showed the distribution of the genes (14,643) and most of the genes were common in all groups (Fig. 6a–c). Kyoto Encyclopaedia of Genes and Genomes (KEGG) functional enrichment analysis showed enrichment in NF-κB, TLR signaling pathway, etc. (Fig. 6d). The cluster heatmap presented differentially expressed genes associated with the TLR signaling pathway (Fig. 6e) where the mixture group presented a higher correlation to the *Am* group than the *Pg* group. Exposure to *Am* with or without *Pg* activated most of the related genes in the TLR signaling pathway which is presented as red. In this study, the gene expression levels of TLR2, MYD88, IKBIP, NFKB1, and C5AR were also assessed and presented (Fig. 6f, g). *Am* participated in the TLR2 modulation and increased gene expression of MYD88 and NFKB1 while simultaneously decreasing the expression of C5AR.

## Discussion

*Am* has garnered attention in periodontal disease treatment due to its microbe-modifying and immune-regulating effects demonstrated in various diseases. In this study, we established a mouse model to investigate the role of *Am* during the host’s exposure to *Pg*. We found that *Am* alleviated *Pg*-induced periodontal inflammation, which was accompanied by an alteration in the periodontal microbial community. Additionally, *Am* enhanced the phagocytosis of THP- 1 derived macrophages, a process mediated by MyD88.

During periodontitis, *Pg* is the key pathogen that impairs the host immune response, evades the immune surveillance, and induces periodontal dysbiosis, leading to further inflammation and tissue destruction [8, 29–33]. Commensals in dental plaque continuously stimulate the orchestrated innate immune response, which can resist the colonization of perio-pathogens. This provides a basis for addressing *Pg* through biological therapy. Accumulating evidence indicates that *Am* benefits the host through modulating host immunity.

As a Gram-negative bacterium, numerous studies indicated that supplementation with *Am*, its pasteurized form, or its extracted protein (Amuc_1100) could activate TLR pathways [34, 35]. These studies demonstrated that *Am* could activate NF-κB and the NLRP3 inflammasome through TLRs, facilitating M1 macrophage polarization [36–38]. These characteristics endow *Am* with potential anti-tumor and anti-infection properties. In accordance with these findings, we observed that *Am* upregulated the transcriptional expression of TLR2 in THP- 1 derived macrophages in vitro, activated the NF-κB pathway, and promoted the production of inflammatory cytokines, particularly chemokines.

However, other studies indicated *Am*’s capacity in suppressing host inflammatory response. For instance, many studies indicate that *Am* can ameliorate colitis [38]. Threonyl-tRNA synthetase secreted by *Am* (*Am*TARS) was reported to restore macrophage homeostasis through specific TLR2 interaction, activating MAPK and PI3 K/AKT pathways to enhance anti-inflammatory IL- 10 production, which ultimately attenuates colitis in mice [39]. Similarly, *Am* was reported to be able to alleviate periodontal inflammation and reduce periodontal bone destruction induced by *Pg* or *Fusobacterium nucleatum* (*Fn*) [15–17, 40]. These studies indicated that *Am* could suppress the virulence of *Pg* and *Fn* and enhance epithelial integrity to improve periodontal condition. Additionally, *Am* was reported to inhibit TLR/MyD88/NF-κB pathway in gingival epithelial cells to reduce inflammation which contrasts with our results where the *Am* upregulate the expression of MyD88 in THP- 1 macrophage. The difference might arise from the dynamic activation of NF-κB pathway that the signal transduction through MyD88 with activated TLRs is transient [41]. In this study, although we did not observe the immune suppression effect of *Am* in vitro, the oral administration of *Am* was found to reduce the expression of IL- 1 and IL- 6 in gingival tissues and decreased alveolar bone resorption induced by *Pg*. The conflicting results between in vitro and in vivo studies may be attributed to *Am* initially triggering a milder and more confined inflammatory response at infected sites to recruit remote immune cells[42, 43]. This process further employs immune cells’ defensive mechanisms to control pathogens and prevent pathogen-induced microbial dysbiosis, thereby inhibiting the amplification of periodontal inflammation. This process is supported by our observations that *Am* activated phagocytosis activity of THP- 1 derived macrophage in vitro, decreased the microbial load in periodontal area, and modified the structure of microbial community in vivo. The reduced inflammation might result from achieving microbial homeostasis.

MyD88 is essential in the TLR signaling pathways, which is important in recruiting immune cells, enhancing phagocytosis, and promoting bacterial eradication by immune cells. TLR/MyD88 dependent signals were reported to enhance the macrophage function, particularly phagocytosis [44–46]. MyD88-deficiency has been shown to dampen the host protective mechanisms [47]. It has been reported that *Pg* manipulates the crosstalk between TLR2 and C5aR leading to the degradation of MyD88 further subverting the host immunity [8]. In this study, *Am* upregulated MyD88 expression in THP- 1 cells, facilitating the internalization of *Pg*. RNA sequencing results showed that *Am* increased TLR2 expression and decreased C5aR expression. Additionally, western blot analysis revealed that *Am* downregulated C5aR protein levels in THP- 1 cells. These findings suggest that *Am* combats *Pg* by interfering with the TLR2/C5aR interplay and activating MyD88, thereby alleviating periodontitis.

A study reported that priming neutrophils with pro-inflammatory cytokines like TNF-α and GM-CSF could reduce the expression of C5aR, suggesting a potential feedback mechanism to prevent excessive inflammation [48]. Interestingly, although the lipid form of *Am*’s cell membrane acts as a TLR2 agonist, it is significantly less potent than conventional agonists [35]. This selective activation pattern suggests *Am* may possess the unique ability to recalibrate immune activation thresholds, potentially explaining its capacity to mount defensive responses without triggering uncontrolled inflammation. These findings gain additional significance when considered alongside recent work demonstrating *Am*’s ability to expand colonic Treg populations and enhance their IL- 10 production in murine colitis models [49]. Although our study observed *Am*’s regulation of C5aR and TLR2 and the downstream cytokine production, the underlying mechanisms require further investigation. Specifically, further research is warranted to verify the TLR2-C5aR-MyD88 pathway in vivo. Furthermore, future studies should investigate whether *Am* mediates its periodontal benefits through alternative mechanisms, including IL- 10 production and regulatory T cell induction. Together, these findings suggest *Am* functions as a immunomodulator through multiple complementary mechanisms: (1) initiating measured pro-inflammatory responses via TLR2/C5aR/MyD88 signaling to combat pathogens, (2) avoiding inflammation amplification through selective receptor activation and cytokine induction, and (3) potentially engaging regulatory pathways (including Treg induction) to restore homeostasis. This confined inflammatory response not only supports *Am*’s safety in clinical applications but also suggests its potential to enhance immune surveillance, thereby preventing dysbiosis at early stages of gingivitis or periodontitis. By modulating the periodontal microbiota before pathogenic communities dominate, *Am* could serve as a preventive or early-intervention strategy. However, its immunogenic properties in severely inflamed periodontal tissues—where immune tolerance may be compromised—require further investigation to determine whethe*r Am* exacerbates or resolves inflammation in advanced disease.

In conclusion, this study highlights the potential of *Am* to act as an immune modulator, restoring the bacterial clearance capacity disarmed by *Pg* and ultimately alleviating periodontitis. These findings offer promising insights into the potential of *Am* as a management option for periodontal disease.

## Supplementary Information

Below is the link to the electronic supplementary material.

Supplementary file1 (DOC 971 KB)

## Data Availability

Sequence data that support the findings of this study have been deposited in NCBI Sequence Read Archive (SRA) under the accession numbers [PRJNA1149924 and PRJNA1150382].
