# Supplementary material for: Akkermansia muciniphila Alleviates Porphyromonas gingivalis-induced Periodontal Disease by Enhancing Bacterial Clearance
Source: Probiotics Antimicrob Proteins. 2025 Apr 29;18(1):666–80. doi: 10.1007/s12602-025-10541-2 (PMC12999801; doi:10.1007/s12602-025-10541-2)
Supplement: Supplementary file 1 — Supplementary file1 (DOC 971 KB) [file 12602_2025_10541_MOESM1_ESM.doc]

*Akkermansia muciniphila* Alleviates *Porphyromonas gingivalis-*Induced Periodontal Disease by Enhancing Bacterial Clearance

Authors

Qin Hu^1^, Wai Keung Leung^1^, Aneesha Acharya^1, 2^, Xuan Li^1*^, and George Pelekos^1*^

^1^. Faculty of Dentistry, The University of Hong Kong, 34 Hospital Road, Sai Ying Pun, Hong Kong SAR, China

^2^. Dr D Y Patil Dental College and Hospital, Pune, India

E-mail of corresponding author: george74@hku.hk

TABLE S1. Primers and probes for quantitative real-time RT-PCR.

| Gene | Primer (5’-3’) |
| --- | --- |
| ACTB | CACCATTGGCAATGAGCGGTTC  AGGTCTTTGCGGATGTCCACGT |
| IL-8 | GAGAGTGATTGAGAGTGGACCAC  CACAACCCTCTGCACCCAGTTT |
| CXCL10 | GGTGAGAAGAGATGTCTGAATCC  GTCCATCCTTGGAAGCACTGCA |
| MCP-1 | AGAATCACCAGCAGCAAGTGTCC  TCCTGAACCCACTTCTGCTTGG |
| TLR2 | CTTCACTCAGGAGCAGCAAGCA  ACACCAGTGCTGTCCTGTGACA |
| MYD88 | GAGGCTGAGAAGCCTTTACAGG  GCAGATGAAGGCATCGAAACGC |
| NFKB1 | GCAGCACTACTTCTTGACCACC  TCTGCTCCTGAGCATTGACGTC |
| C5AR | CCATTAGTGCCGACCGTTTCCT  CACGAAGGATGGAATGGTGAGG |


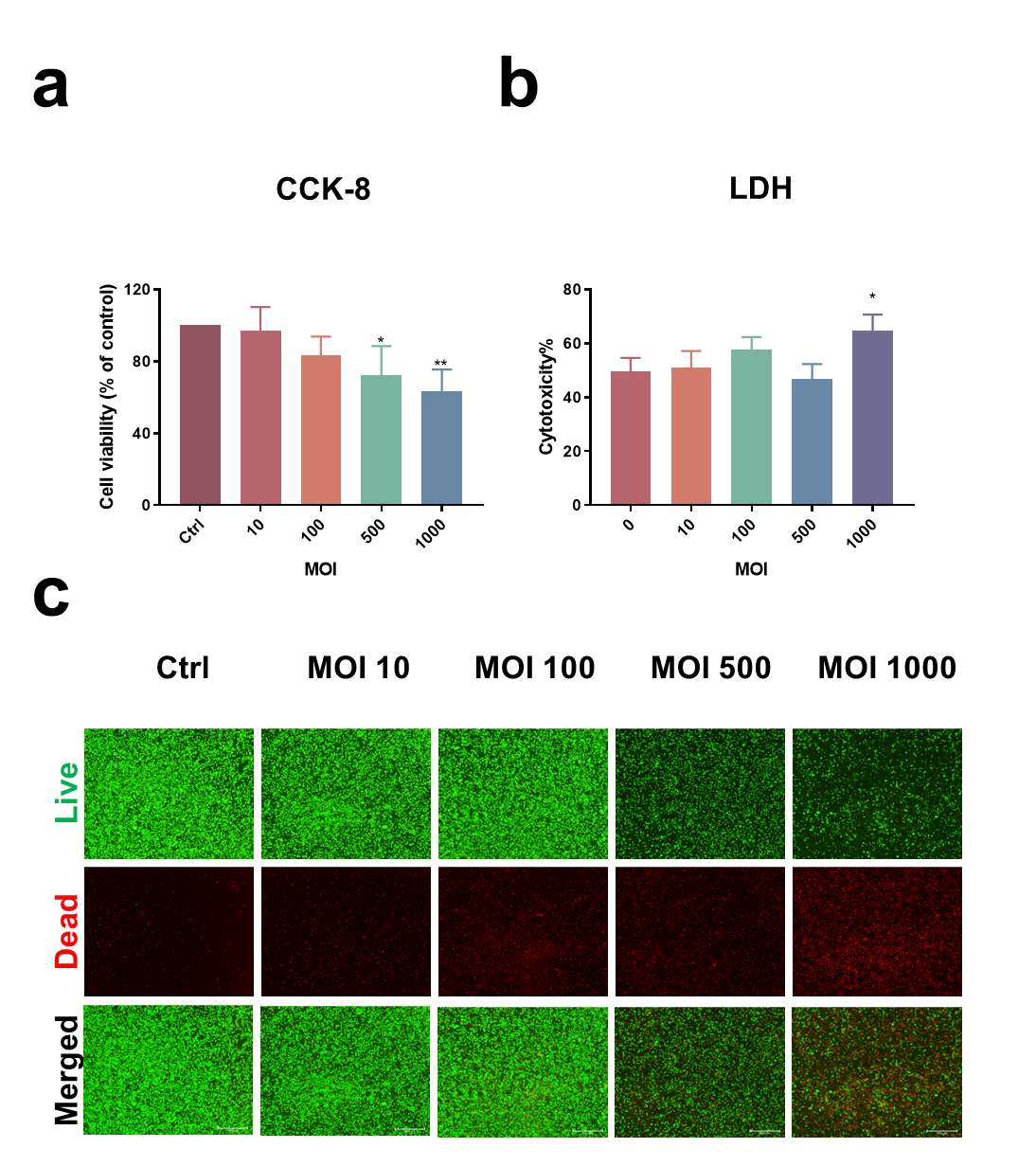


**Fig S1**. **Effects of *Am* in viability and cytotoxicity on THP-1 differentiated macrophages.** (a) Cell viability and (b) relative LDH release were assessed in macrophages exposed to *Am* at infection ratios of 0, 10:1, 100:1, 500:1, and 1000:1 for 24 hours. (c) Live/Dead cell staining was conducted on cells cultured with *Am* for 24 hours in culture medium, with live cells appearing green and dead cells appearing red under fluorescence microscopy (scale bar, 500 µm).
